# Case Report: Long-term surgical outcomes in pug dogs with articular facet dysplasia-associated thoracolumbar myelopathies

**DOI:** 10.3389/fvets.2025.1648444

**Published:** 2025-08-22

**Authors:** Anna Tauro, Colin J. Driver, Jeremy Rose, Ricardo Fernandes, Clare Rusbridge

**Affiliations:** ^1^Fitzpatrick Referrals, Neurology Service, Eashing, United Kingdom; ^2^Department of Clinical Sciences, North Carolina State University, Raleigh, NC, United States; ^3^ACCESS Specialty Animal Hospitals, Pasadena, CA, United States; ^4^Lumbry Park Veterinary Specialists, Alton, United Kingdom; ^5^The Ralph Veterinary Referral Centre, Marlow, United Kingdom; ^6^School of Veterinary Medicine, University of Surrey, Guildford, United Kingdom; ^7^Wear Referrals Veterinary Specialist & Emergency Hospital, Bradbury, United Kingdom

**Keywords:** pug myelopathy, constrictive myelopathy, spinal arachnoid diverticulum, intervertebral disc disease, vertebral stabilization, canine thoracolumbar myelopathy, postsurgical outcome, dog

## Abstract

Pug dogs are predisposed to thoracolumbar myelopathy associated with vertebral articular process dysplasia, suggesting a biomechanical etiology. While surgery is commonly pursued, long-term outcomes remain poorly defined. This retrospective descriptive case series reports on seven Pug dogs that underwent surgical treatment for thoracolumbar myelopathy and were followed up for at least 7 years postoperatively. All dogs were part of a previously published cohort treated at the same institution and were included in this follow-up based on caregiver consent. Dogs were classified into three groups—intervertebral disc protrusion (IVDP), spinal arachnoid diverticulum (SAD), or pia-arachnoid fibrosis (PAF)—based on imaging and intraoperative findings. All dogs underwent vertebral stabilization, with decompression performed in the IVDP and SAD groups. Follow-up data were collected via caregiver questionnaire and review of clinical records. Temporary clinical improvement or stabilization was achieved in five of seven Pug dogs (71%), particularly in the IVDP and SAD groups. Recurrence of neurological signs was noted in four of these five dogs (80%), with a median time to recurrence of 8.9 months. The median survival time following surgery across all seven dogs was 3.2 years. Dogs with PAF demonstrated the poorest outcomes. Favorable outcomes were associated with younger age, shorter lesion extension, and milder T2-weighted hyperintensity. Urinary and/or fecal incontinence and recurrent urinary tract infections were common comorbidities. Surgery was considered beneficial by caregivers in four (57%) of seven cases. In conclusion, vertebral stabilization with decompression may provide temporary clinical benefit in Pug dogs with thoracolumbar myelopathy. However, recurrence is common, and PAF may predict a less favorable prognosis. Larger-scale studies are warranted to explore potential associations between intrathecal inflammation, the necrotizing meningoencephalitis (NME) risk haplotype, and response to adjunctive therapies such as corticosteroids or cerebrospinal fluid diversion techniques. Urinary incontinence should be closely monitored to minimize complications.

## 1 Introduction

Thoracolumbar myelopathy is a common condition in Pug dogs, typically presenting at a median age of 8.5 years (1, 2). It is characterized by a complex disease process, often involving vertebral articular process (VAP) dysplasia, which is suspected to contribute a dynamic compressive component (1, 3, 4). The authors use the term VAP dysplasia instead of the commonly used caudal articular process (CAP) dysplasia, as the malformation may involve either or both cranial and caudal articular processes (1, 5).

Typical clinical signs include chronic spastic paraparesis and pelvic limb proprioceptive ataxia, usually without spinal hyperesthesia. Urinary and/or fecal incontinence may also occur.

The investigation of the disease, which predominantly involves the vertebral region between T10 and L1 (1, 5–7), typically includes both computed tomography (CT) and magnetic resonance imaging (MRI) scans of the thoracolumbar spine. CT confirms the presence of vertebral malformation, while MRI reveals intramedullary hyperintensity on T2-weighted images (T2WI), which is located at the same level as the VAP dysplasia and may be associated with other spinal cord conditions such as spinal arachnoid diverticulum (SAD), intervertebral disc protrusion (IVDP), pia-arachnoid fibrosis (PAF), or a combination of these (1, 5, 8).

There is evidence suggesting that an immunological etiology contributes to myelopathy in Pug dogs (9, 10). Chronic spinal cord insults, such as vertebral dynamic instability or disc herniations, may lead to lymphohistiocytic inflammation in the central nervous system and cerebrospinal fluid (CSF), secondary to astrocyte activation. This type of inflammation is also a predominant feature of necrotizing meningoencephalitis (NME) in this breed. Astrocyte activation results in increased CSF levels of glial fibrillary acidic protein (GFAP) and may trigger the production of anti-GFAP autoantibodies in affected Pug dogs (9, 10). While the pathogenicity of these autoantibodies toward astrocytes remains unclear, it is hypothesized that they may cause astrocytopathy, contributing to a chronic proliferative state characterized by fibrosis and permanent adhesions (10).

The optimal treatment and long-term management of Pug myelopathy remain uncertain. However, some studies suggest that decompressive laminectomy combined with vertebral stabilization may lead to neurological improvement in affected dogs (1, 4).

In the authors’ previous VCOT Open publication in 2019, titled *Surgical Management of Thoracolumbar Myelopathies in Pug Dogs with Concurrent Articular Facet Dysplasia*, vertebral stabilization—either alone or combined with spinal cord decompression—was shown to potentially halt the progression of, or improve, clinical signs in Pug dogs with thoracolumbar myelopathy. That study focused on short- and mid-term outcomes, with follow-up evaluations conducted through re-examinations and phone interviews up to 2 years post-surgery.

This current study builds on that work, offering the first long-term follow-up of these same cases, covering a period of more than 7 years after the initial surgical interventions in 2017. It aims to provide new insights into the long-term effectiveness of surgical management and its impact on neurological outcomes and quality of life in Pug dogs with thoracolumbar myelopathies.

## 2 Materials and methods

This descriptive case series builds on the authors’ 2019 *VCOT Open* publication, which described the surgical interventions performed on Pug dogs treated at Fitzpatrick Referrals, Surrey, United Kingdom in 2017 (Table 1).

**Table 1:** Follow-up data on vertebral stabilization outcomes in dogs with Pug myelopathy, including clinical progression, recurrence, urinary complications, and overall surgical benefit.

Dog	Clinical signs duration (months)	Age at 1^st^ surgery (years)	1^st^surgical intervention	Time in- between surgeries (months)	TOTAL clinical signs duration prior to STAB (months)	Age at STAB (years)	Date of STAB	Surgical intervention with STAB	Post-STAB functional grading (0–4)	Time to recurrence after STAB (months)	Age of death (years)	Alive (age)	Incontinence (U/F)			UTI	Urinary tract surgery	Overall outcome (surgical benefit)
													Pre-STAB	Post-STAB	Follow-up			
1					0.8	6.9	July 2017	IVDP	4	47.2	11.9		F	F	U	Y		Y
3	5.5	9.1	dLM + SAD	17.8	23.2	10.5	April 2017	IVDP	3	11.3	13.1				U	Y		Y
6					9	8.9	June 2017	PAF	0 (in dog cart)	5.4	15.9		F	U/F	U/F	Y	Permanent cystotomy catheter	N
9					0.7	5	March 2017	PAF	2	6.4	6.4*			F	U/F			N
11					30	9.2	Jan 2017	PAF / SAD	0	0	9.5*		U/F	U/F	U/F	Y		N
12					15	9	March 2017	SAD	3	27.1	12.1		F					Y
13	5.5	1.4	HL + SAD	6.9	12.3	2	May 2017	SAD	4		ALIVE	9.2	U/F					Y
Med	5.5				12.3	8.9				8.9	12.1							

*Spinal condition was the cause of death. dLM, dorsal laminectomy; F, fecal; HL, hemilaminectomy; IVDP, intervertebral disc protrusion; Med, median; PAF, pia-arachnoid fibrosis; N, no; SAD, spinal arachnoid; diverticulum; STAB, stabilization; U, urinary; UTI, urinary tract infection; Y, yes.

Dog caregivers were contacted via phone and/or email in the second half of 2024 to complete a follow-up questionnaire and provide consent for retrieving clinical records.

The questionnaire collected data on post-surgical mobility grading, recurrence of neurological signs, presence of urinary and/or fecal incontinence, cause of death (if applicable), and overall post-surgical outcomes.

In the authors’ previous paper (1), all Pug dogs exhibited moderate paraparesis and ataxia prior to surgical intervention. In the present study, post-stabilization mobility is compared to pre-surgical status and graded on a 0 to 4 scale (Table 2):

**Table 2:** Functional grading scale.

Functional grading	
4	Optimal functional ambulation
3	Moderate functional ambulation
2	Mild functional ambulation
1	No change
0	Deterioration

This table shows the functional grading scale for assessing ambulation in patients.

**Grade 0**: ‘Deterioration’—worsening motor function or complete loss of mobility.

**Grade 1**: ‘No change’—no visible improvement.

**Grade 2**: ‘Mild functional recovery’—slight improvement, such as the ability to stand or take at least 10 steps without assistance, though severe paraparesis and ataxia persisted. Significant support was still required for longer walks.

**Grade 3**: ‘Moderate functional recovery’—noticeable improvement, with the dog able to walk unassisted, though residual weakness or incoordination persisted.

**Grade 4**: ‘Optimal functional recovery’—near or complete return to pre-injury motor function, allowing the dog to walk, run, and perform normal activities with minimal or no neurological deficits.

Post-surgical outcome was evaluated from the caregivers’ perspective, focusing on the dog’s quality of life and neurological recovery relative to its pre-surgical status. This was assessed through a binary response, where caregivers indicated whether the surgery was perceived as beneficial with a ‘yes’ or ‘no’ answer.

Clinical histories were reviewed for details on sex, neutered status, age (in years) at stabilization surgery, whether other spinal surgeries had been previously performed for the same neurological condition, neurological signs duration, time to recurrence, follow-up imaging (if applicable), age and cause of death (if applicable), presence of urinary and/or fecal incontinence, and urinary tract infections (UTIs). Additionally, results from genetic testing for degenerative myelopathy (DM), CSF analysis, and serology for infectious diseases, when available, were also recorded. Following a recent study (10) suggesting a potential association between NME and Pug myelopathy, we checked for NME genetic testing, but none of the dogs had been tested.

**Figure 1:**
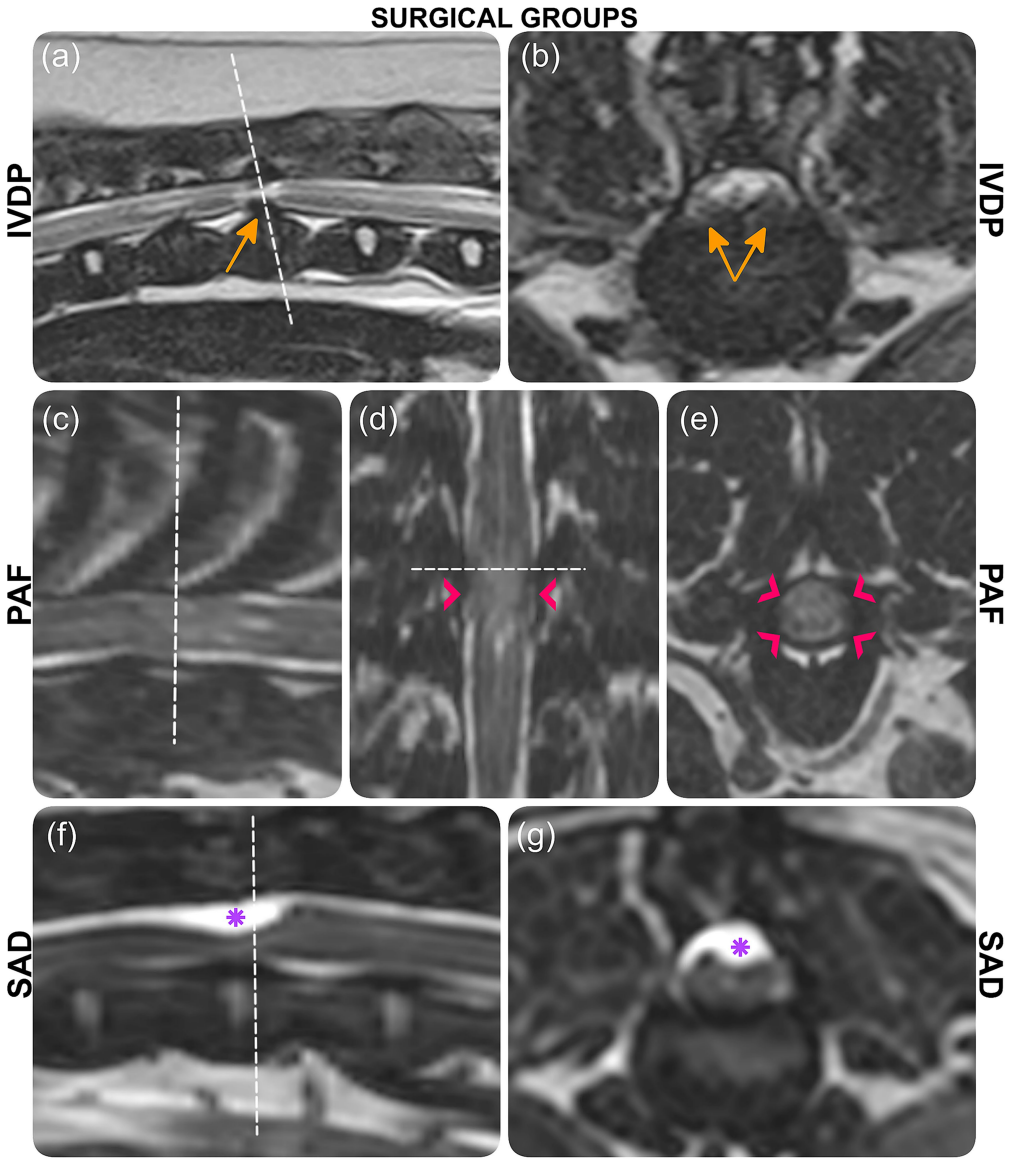
MRI images depicting three surgical groups: IVDP (case 1), PAF (case 6), and SAD (case 13). **(a, b)** T2-weighted images with orange arrows indicating disc protrusion. **(c, e)** 3D-CISS images with pink arrowheads marking pia-arachnoid bands and CSF signal disruption. **(f, g)** 3D-CISS images with purple asterisks highlighting the arachnoid diverticulum. Each section is labeled accordingly.

To ensure continuity with the previously published study, cases were assigned the same cardinal numbers and grouped by the same surgical classifications: intervertebral disc protrusion (IVDP), spinal arachnoid diverticulum (SAD), and pia-arachnoid fibrosis (PAF; Figure 1).

Factors potentially influencing outcomes, including the operating surgeon, type of surgery, stabilization construct, spinal cord signal intensity, surgical group, age at stabilization, prior spinal surgeries, neurological signs duration, DM results, CSF analysis, and history of incontinence or UTIs, were assessed.

Details regarding the surgical techniques associated with vertebral stabilization were reported, including the type of laminectomy performed. In SAD cases, it was noted whether a dural substitute was used following durotomy and marsupialization. The characteristics of the vertebral constructs were documented, noting whether stabilization was unilateral or bilateral, the number of vertebrae involved, and any evidence of pin lucency or vertebral instability observed on follow-up CT imaging (when performed).

Pre-surgical magnetic resonance (MR) images in T2-weighted (T2W) sagittal and transverse planes were reviewed by the author (AT) to assess whether spinal cord signal intensity at the lesion level, associated with VAP dysplasia, influenced the outcome. The analysis included documenting the location, grading, and extent of spinal cord hyperintensity.

Location was classified as predominantly affecting gray matter, white matter, or both. Grading was based on brightness relative to gray matter and CSF: mild (slightly brighter than gray matter), moderate (brighter than gray matter but less intense than CSF), or severe (matching CSF intensity). The extent of hyperintensity was measured on the sagittal plane and expressed as the ratio of hyperintensity length to L2 vertebral body length (11).

Additionally, the T2W hyperintensity area was examined on T1-weighted (T1W) transverse images (when available), with signal intensity categorized as iso-, hyper-, or hypointense relative to gray matter (12).

Due to the limited sample size, statistical analyses were not performed, and results are presented descriptively. All dogs received in-clinic postoperative physiotherapy; however, due to variability in protocols and limited follow-up—especially when care was sought externally—physiotherapy was not evaluated as a factor influencing outcome.

## 3 Results

### 3.1 Case details and surgical procedures

As described in the authors’ prior report (1), 14 Pug dogs underwent vertebral stabilization surgery. One dog (case 4) was euthanized within a year due to an unrelated oral sarcoma. As this case could not be assessed for long-term neurologic outcome, it was excluded from the current analysis.

Of the remaining 13 dogs, caregivers for seven dogs consented to participate in this follow-up study (Tables 1, 3). These included 2 neutered females (cases 1 and 13), 3 neutered males (cases 3, 6, and 12), and 2 entire males (cases 9 and 11). Male dogs were overrepresented, consistent with findings in our previous study. However, cases 3 and 13 had been neutered since the previous study, leading to a slight overrepresentation of neutered male dogs within this subset of the population. The median age of the seven dogs at the time of stabilization was 8.9 years (range: 2 to 10.5 years), with median duration of clinical signs prior to stabilization of 12.3 months (range: 0.7 to 30 months).

**Table 3:** Details on surgical groups, sex and neuter status, operating surgeon, surgeries performed, stabilization constructs, spinal cord intensity, follow-up CT, DM genetic testing, CSF analysis, serology results, and cause of death.

Dog	Groups	Sex	Surgeon	Surgeries associated with STAB	Stabilization construct			Spinal cord hyperintensity on T2WI			Intensity on T1WI	Follow-up CT (months from STAB)	Construct stability failure	DM Testing (SOD1 mutation)	CSF analysis			Serology		Concurrent disorders	Cause of death
					# Vertebrae	Unilateral	Bilateral	Site	length/L2 (mm)	Grade					SITE	Protein (mg/dL)	TNCC (/uL)	*Toxoplasma gondii*	*Neospora caninum*		
1	IVDP	FN	JR	mHL + LPC/pD	4	Y		GM	1.11	S					C	39	4	negative	negative		IBD / PLE = PTS
3	IVDP	MN	CJD	mHL + LPC/pD	3	Y		GM + WM	1.44	Mo											BOAS / tracheal collapse = PTS
6	PAF	MN	CJD		4		Y	GM + WM	2.22	Mo		1.8		negative							Seizure
9	PAF	ME	JR		4	Y		GM	1.33	S	ISO	7.1	Y	carrier	L	116	14	negative	negative		Spinal deterioration = PTS
11	SAD (+PAF)	ME	CJD	dLM + M	3	Y		GM	0.84	S	ISO									T1-T5 SM Stromal ulcer	Spinal deterioration = PTS
12	SAD	MN	CJD	dLM + M	2	Y		GM + WM	3.50	Mo		7.0									DOA (organ failure)
13	SAD	FN	CJD	dLM + M (+dural graft)	4	Y		GM + WM	0.88	Mi											ALIVE

BOAS, brachycephalic obstructive airway syndrome; C, cerebellomedullary cistern; CJD, Colin J Driver; CT, computed tomography; dLM, dorsal laminectomy; DM, degenerative myelopathy; DOA, death on arrival; FN, female neutered; IBD, inflammatory bowel disease; ISO, isointense; IVDP, intervertebral disc protrusion; JR, Jeremy Rose; L, lumbar cistern; LPC, lateral partial corpectomy; GM, gray matter; GM+WM, gray and white matter; M, durotomy and marsupialization; ME, male entire; Mi, mild; Mo, moderate; mHL, mini-hemilaminectomy; MN, male neutered; N, no; PAF, pia arachnoid fibrosis; pD, partial discectomy; PLE, protein losing enteropathy; PTS, put to sleep (euthanized); S, severe; SAD, spinal arachnoid fibrosis; STAB, stabilization; T1WI, T1-weighted images; T2WI, T2-weighted images; TNCC, Total Nucleated Cell Count; W, white matter; Y, yes.

All surgeries were performed by one of two board-certified neurologists (CJD or JR), as indicated in Table 3. Two dogs (cases 3 and 13) underwent spinal surgeries for spinal arachnoid diverticulum (SAD) prior to vertebral stabilization at ages 9.1 years (case 3) and 1.4 years (case 13). In both cases, clinical signs recurred 1.2 months (case 3) and 1.9 months (case 13) after surgery, progressing for 16.6 and 5 months, respectively. Repeated MRI revealed IVDP (case 3) and recurrent SAD (case 13), and spinal CT confirmed the diagnosis of VAP dysplasia in both. Decompressive spinal surgery was performed alongside stabilization surgery at 10.5 years (case 3) and 2 years of age (case 13).

All dogs underwent vertebral stabilization using IMEX™ miniature threaded interface pins encased in tobramycin-impregnated polymethylmethacrylate (PMMA) bone cement. In addition to stabilization, a mini-hemilaminectomy, partial corpectomy, and partial discectomy were performed to address IVDP in cases 1 and 3. It is important to note that the partial corpectomy and partial discectomy were performed using a pneumatic drill, as described by Crawford (13). This created a window through the caudal vertebral endplates and the affected intervertebral disc, while leaving the most dorsal part of the anulus fibrosus intact. This dorsal section was then pushed ventrally, which was observed intraoperatively to relieve spinal cord compression.

For SAD cases (11, 12, and 13), dorsal laminectomy, durotomy, and marsupialization were performed in addition to stabilization. In case 13, BioSIS™—a dural substitute derived from porcine small intestinal submucosa (SIS)—was used to overlay the dural defect, based on the surgeon’s preference. Retrospective MRI review by the author (AT) also identified concurrent PAF in case 11, classifying this dog within both the SAD and PAF groups. Vertebral stabilization alone was performed in cases 6 and 9, which were affected by PAF without compressive lesions.

### 3.2 DM, CSF analysis, serology for infectious diseases test results

The decision to perform genetic testing for DM, CSF analysis, or serology for infectious diseases was at the discretion of the surgeon (Table 3).

Genetic testing for DM was performed by Laboklin® (Manchester, United Kingdom) in two cases: case 6 was negative, and case 9 was a carrier of one SOD1 mutated copy.

CSF was collected prior to surgical stabilization from the cerebellomedullary cistern in case 1 and the lumbar cistern in case 9 and sent for analysis to TDDS® Labs (Exeter, UK), which is now known as Veterinary Pathology Group (VPG). Case 1 showed a slight increase in protein levels (39 mg/dL; reference range <25), with a normal nucleated cell count (NCC), consistent with albuminocytologic dissociation. In contrast, case 9 showed an increase in both protein (116 mg/dL; reference range <45) and NCC (14 cells/μL; reference range <5 cells/μL). Cellularity consisted of 72% small lymphocytes, 23% monocytes, 3% non-degenerated neutrophils, and 2% eosinophils, compatible with mild mononuclear pleocytosis. No overt neoplastic or infectious agents were observed.

Serology for *Toxoplasma gondii* and *Neospora caninum* was performed by Veterinary Pathology Group (VPG), for cases 1 and 9, with both results returning negative.

### 3.3 Imaging findings and stabilization constructs

**Figure 2:**
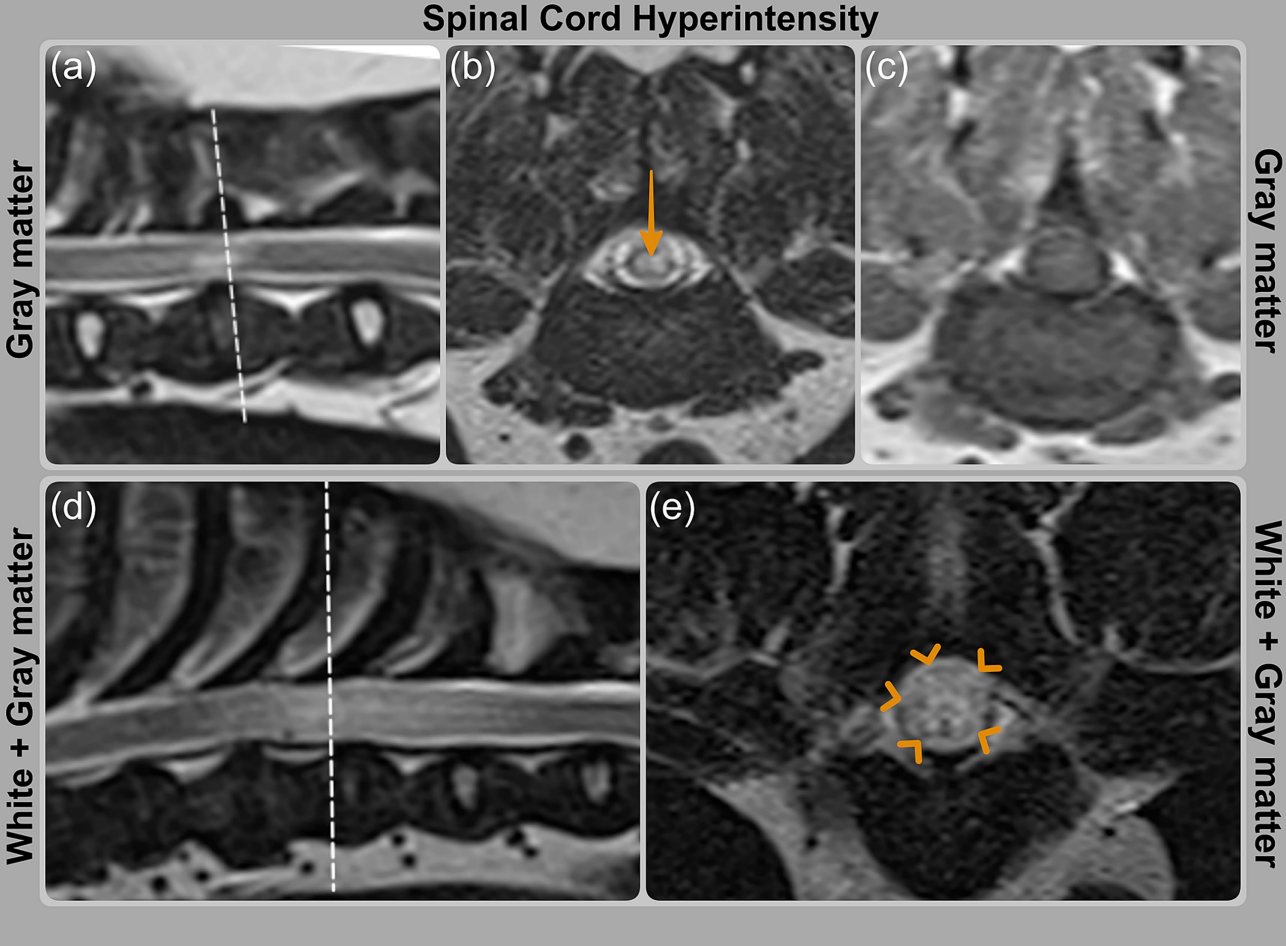
MRI images showing spinal cord signal intensity grading on T2WI. **(a, b)** Case 9 (PAF) with orange arrow indicating hyperintensity predominantly affecting gray matter; **(c)** corresponding T1WI image is isointense. **(d, e)** Case 6 (PAF) with hyperintensity involving both white and gray matter, highlighted by orange arrowheads.

MR imaging was performed using a 1.5 T scanner (Siemens Symphony Tim system, Erlangen, Germany) prior to stabilization surgery. The images were reviewed and revealed that spinal cord signal intensity predominantly affected the gray matter in three cases (cases 1, 9, and 11) and both, gray and white matter, in four cases (cases 3, 6, 12 and 13; Table 3; Figure 2). The signal intensity was graded as mild in one case (cases 13), moderate in three cases (cases 3, 6 and 12), and severe in three cases (cases 1, 9, and 11). The longest T2-hyperintensity: L2 ratio was seen in case 12, followed by cases 6, 3, 9, 1, 13 and 11 (Table 3). T1W transverse images were available for only two cases (9 and 11), in which the areas of T2W hyperintensity appeared isointense to gray matter on T1W sequences.

Postoperative computed tomography (CT) scans were performed on all dogs using a 160-slice scanner (Aquilion Prime™, Toshiba, Otawara, Japan). Follow-up CT scans were conducted in cases 6, 9, and 12 at 1.8, 7.1, and 7 months post-stabilization surgery, respectively. Radiolucency around one pin in the caudal vertebra (T13) of the four-vertebra construct (T10–T13) was observed exclusively in case 9, accompanied by mild increased space between the cement–bone interface and multifocal osteolysis in the vertebral bone surrounding the cement. Further investigations were declined by the owner.

The stabilization construct was unilateral in six cases and bilateral in case 6. Constructs involved two vertebrae in case 12, three vertebrae in cases 3 and 11, and four vertebrae in the remaining four cases (cases 1, 6, 9, and 13; Table 3).

### 3.4 Fecal and urinary incontinence

Of the seven dogs, five (71%)—cases 1, 3, 6, 9, and 11—exhibited urinary and/or fecal incontinence, and four of these (80%)—cases 1, 3, 6, and 11—experienced recurrent urinary tract infections (UTIs) following stabilization surgery (Table 1).

Case 3 had recurrent UTIs diagnosed using in-house testing, including urine dipstick and microscopic sediment analysis, which revealed blood, bacteria, and leukocytes.

Urine cultures were performed in four cases (1, 6, 9, and 11). Case 9 yielded a negative result, while *Escherichia coli (E. coli)* was isolated in cases 1, 6, and 11. In case 6, subsequent cultures grew *coagulase-positive Staphylococcus*, *Proteus* spp., *Serratia* spp., and *Pseudomonas aeruginosa*. In case 11, later culture yielded a mixed growth of *Staphylococcus pseudintermedius* and *E. coli*.

All four dogs diagnosed with UTIs (cases 1, 3, 6, and 11) were initially treated with potentiated amoxicillin (amoxicillin-clavulanate). However, urine sensitivity testing in cases 1 and 6 revealed increasing antibiotic resistance, requiring a switch to alternative treatments including enrofloxacin, marbofloxacin, doxycycline, and trimethoprim/sulfamethoxazole. In cases 1, 3, and 6, the rDVMs did not specify the type of urinary incontinence. In case 3, the rDVM recommended medications for urethral sphincter mechanism incompetence, such as phenylpropanolamine or estriol, but these were not prescribed. Case 6 was initially treated with phenylpropanolamine but was later diagnosed with an upper motor neuron bladder secondary to thoracolumbar myelopathy. Treatment was subsequently adjusted to include prazosin, bethanechol, and either manual bladder expression or catheterization. Unfortunately, 13.1 months after stabilization, Case 6 developed a urethral obstruction caused by multiple uroliths associated with an *E. coli* urinary tract infection. Treatment included retrograde flushing, percutaneous cystolithotomy, and placement of a permanent cystostomy catheter to manage detrusor atony. Urolith analysis revealed a mixed composition of calcium-based minerals and struvite.

In cases 9 and 11, urinary retention was diagnosed secondary to thoracolumbar myelopathy at our referral hospital, but no specific treatment was initiated.

### 3.5 Functional recovery post-stabilization surgery across surgical groups

Of the seven cases, five dogs (71%)—cases 1, 3, 9, 12, and 13—regained functional ambulation postoperatively, with recovery grades ranging from 2 to 4 (Tables 1, 2). Among these five, four dogs (80%)—cases 1, 3, 9, and 12—experienced recurrence of neurological signs. Case 13, from the SAD group, was nearly 4 years younger than the others at the time of presentation and remains neurologically stable and ambulatory at 9.2 years of age—the only dog still alive at the time of writing.

The median time to recurrence across all seven dogs was 8.9 months (range: 0–47.2 months). Dogs in the PAF group (cases 6, 9, and 11) exhibited the least favorable outcomes. The extended survival of case 6, despite short-lived neurological recovery, was facilitated by the use of a dog mobility cart.

Case 11, which had both PAF and SAD, was also diagnosed with T1–T5 syringomyelia and later underwent enucleation due to a severe corneal ulcer—both factors that may have contributed to the worsening in the postoperative period and failure to improve (1; Tables 1, 2).

### 3.6 Lifespan, cause of death, and overall outcome

Six dogs (cases 1, 3, 6, 9, and 12) died at a median age of 12.1 years (range: 6.4–15.9 years), with a median survival time of 3.2 years following vertebral stabilization (Table 1). Two cases—cases 9 and 11—were euthanized due to a spinal-related cause; the remaining dogs died of unrelated causes: inflammatory bowel disease with protein-losing enteropathy (case 1), brachycephalic obstructive airway syndrome (case 3), seizures (case 6), and organ failure (case 12; Table 3). Case 13 is currently alive at 9.2 years of age. From the caregivers’ perspective, the overall post-surgical outcome was considered beneficial in four of seven cases (57%; 1, 3, 12, and 13).

## 4 Discussion

### 4.1 Is vertebral stabilization beneficial in pug myelopathy?

This study suggests that vertebral stabilization, combined with decompressive procedures (IVDP and SAD groups), may have contributed to temporary clinical improvement and delayed recurrence of signs in five of the seven dogs (71%; cases 1, 3, 9, 12, and 13).

Given the small sample size and absence of a control group, these findings should be interpreted with caution. The median time to recurrence of neurological signs among the improved cases was 8.9 months (range: 0–47.2 months; Table 1).

SAD group had the most favorable survival outcomes, although case 13 was notably younger at the time of surgery. In contrast, PAF group exhibited the least favorable prognosis, with the shortest time to recurrence. All three dogs in this group—cases 6, 9, and 11—had poor long-term outcomes: cases 9 and 11 were euthanized due to progression of the spinal condition, with case 11 showing immediate postoperative deterioration—an outcome that highlights the potential for acute decline following complex spinal surgery—and case 6 ultimately required a mobility cart (Tables 1, 2).

According to caregiver evaluations, the surgery was perceived as beneficial in four out of seven cases (57%).

No differences in clinical outcome were observed between the two surgeons. In the IVDP group, a mini-hemilaminectomy and partial discectomy were used to address compressive disc protrusion, a technique known to reduce spinal cord manipulation and post-surgical complications (13) (Table 3).

Previous studies on SAD have not determined the best surgical approach. Reported techniques include durotomy, durectomy, marsupialization, shunt placement, and vertebral stabilization. Earlier studies (14, 15) were published before VAP dysplasia was recognized as a clinical entity in Pug dogs and may not have excluded it, despite using spinal CT. More recent studies (16–19) often lacked spinal CT confirmation of VAP dysplasia and may have underdiagnosed dynamic instability. While recurrence of signs typically occurs months to years after surgery (16, 17, 20), shorter follow-up studies (18) may miss these late relapses. A recent study by Mól (20) diagnosed VAP dysplasia using spinal CT but did not to perform vertebral stabilization. Instead, it proposed that using 10x surgical magnification and Durepair™ —a bovine pericardium-based dural substitute—may have reduced recurrence by facilitating more precise removal of leptomeningeal adhesions. Gomes (19) proposed an unsutured subdural shunt (SDS) technique for SAD, reporting improved long-term outcomes. However, Pug dogs—who had the highest recurrence rates—were not assessed for VAP dysplasia or NME, both of which may be contributing factor.

Overall, Pug dogs appear to have the poorest long-term outcomes across published studies (16, 17). Furthermore, the absence of follow-up MRI in several reports (18–20) introduces uncertainty as to whether the recurrence of clinical signs was due solely to SAD.

In our study, vertebral stabilization was considered essential, as both cases 3 and 13 experienced recurrence following decompressive surgery alone. Case 3 relapsed at 17.8 months and case 13 at 6.9 months. Similar outcomes have been reported by Alisauskaite (17), suggesting that early clinical improvement may be transient in such cases. These findings emphasize the need for long-term follow-up and support the use of spinal CT for identifying VAP dysplasia (1).

**Figure 3:**
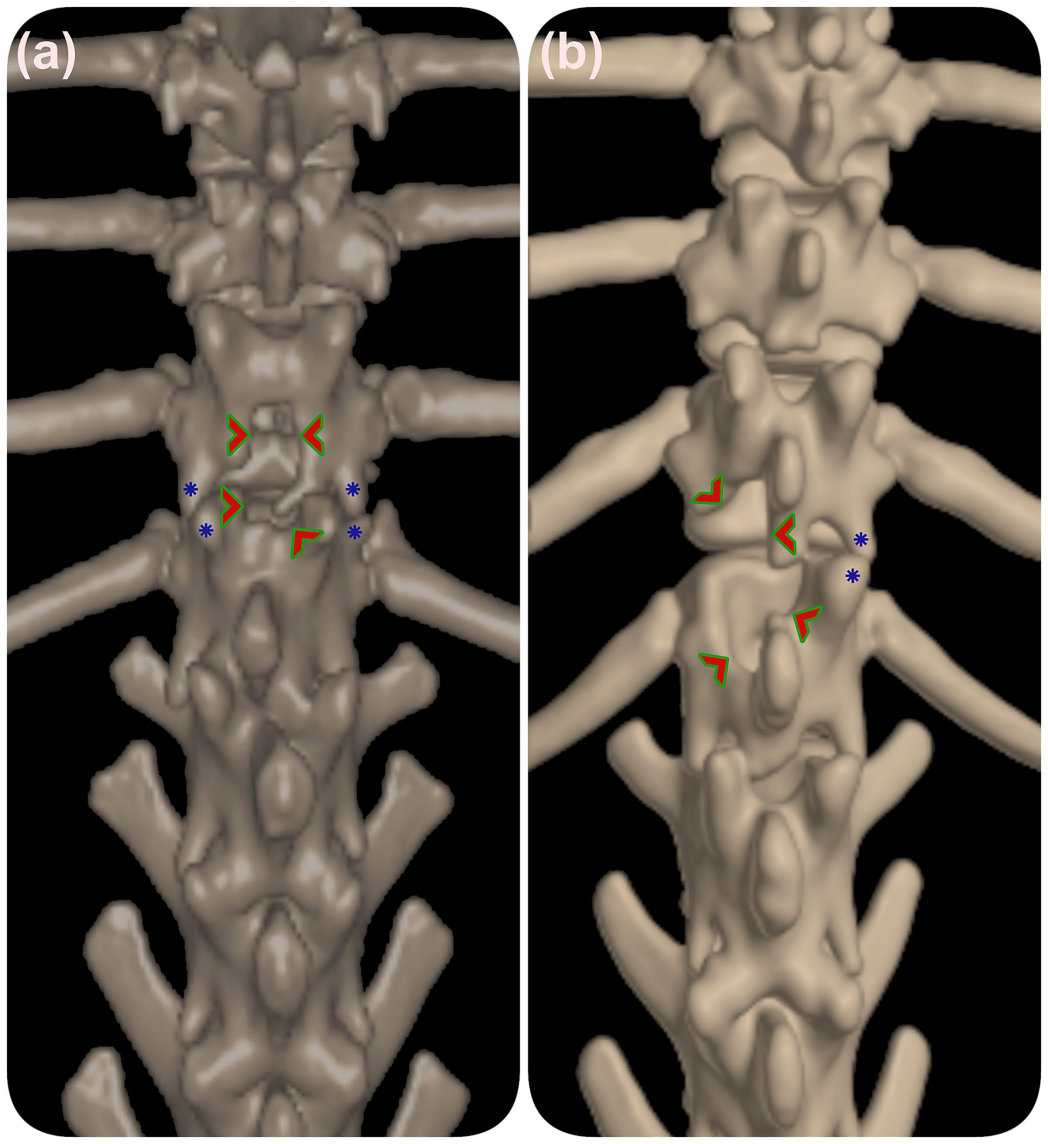
3D reconstructed CT of the thoracolumbar spine prior to vertebral stabilization. **(a)** Dorsal laminectomy in case 3 and **(b)** hemilaminectomy in case 13 are indicated by green arrowheads. Blue stars mark dysplastic vertebral facets. These images illustrate how prior laminectomy procedures may impact vertebral stability in relation to facet malformations.

Performing laminectomies without stabilization can exacerbate dynamic instability, particularly in small breeds like Pugs. In such cases, laminectomies may inadvertently damage the VAPs, as seen in cases 3 and 13 (Figure 3), likely contributing to recurrence.

We chose marsupialization for its previously reported favorable outcomes (14). Although 2.5x magnification was used in our surgeries, this does not offer the same level of precision as a surgical microscope (20), which may improve the removal of leptomeningeal adhesions. The SDS technique (19) may be a promising option for future cases, particularly in PAF cases, as it may help reestablish CSF flow. In one case (case 13), BioSIS™, a porcine-derived dural substitute, was used. This may offer advantages over bovine-derived substitutes due to its low rate of adverse reactions (20, 21).

### 4.2 Do dogs in the PAF group have a worse prognosis? (this part of the text discusses the immunological contribution to the development of pug myelopathy)

Dogs in the PAF group exhibited only transient improvement following surgery and had the poorest overall prognosis. Case 6 eventually required a mobility cart, while cases 9 and 11 were euthanized due to progressive neurological decline (Table 3). Follow-up CT imaging in case 9 revealed mechanical failure of the implant, which may have contributed to the deterioration. Dynamic imaging techniques, such as flexion-extension radiographs or dynamic CT, were not performed, limiting assessment of vertebral instability. In case 11, clinical deterioration was likely multifactorial, with concurrent T1–T5 syringomyelia and the subsequent development of a severe corneal ulcer requiring enucleation, both of which may have adversely impacted the post-operative course and overall outcome.

PAF, characterized by pia-arachnoid fibrosis and CSF flow disruption (2, 7–9, 22), is another term for constrictive myelopathy (2, 23, 24). The underlying cause remains unknown, though genetic and inflammatory factors may contribute (9, 10, 25). MRI findings show a focal, circumferential spinal cord lesion (7, 9), with absence of CSF signal within the subarachnoid space on T2W images, thickened pia-arachnoid bands (best visualized on 3D-CISS sequences), and secondary spinal cord edema due to impaired CSF flow (1, 8) (Figures 1c–e).

Although VAP dysplasia is widespread in Pug myelopathy (2), the intramedullary hyperintense spinal cord lesion is typically focal. The authors hypothesized that this focal presentation occur because dynamic instability generates forces most pronounced in areas of high spinal mobility, particularly at the transition between the caudal thoracic and cranial lumbar regions (6). The lesion typically develops where these forces pivot, concentrating mechanical pressure on the spinal cord, even when VAP dysplasia is more widespread.

Vertebral stabilization was selected as the sole surgical intervention for PAF cases (cases 6 and 9). This decision was guided by imaging findings, which revealed circumferential thickening of the pia-arachnoid membranes without a discrete compressive lesion amenable to decompression. In the absence of a clear surgical target, the benefit of more invasive interventions—such as dissection of diffuse subarachnoid adhesions—remains uncertain. Therefore, stabilization alone was performed to reduce vertebral micromotion and slow potential disease progression. Case 11, which had concurrent PAF and SAD, underwent dorsal laminectomy, durotomy, and marsupialization—similar to other SAD cases—in addition to vertebral stabilization.

Given the poor long-term outcomes observed, alternative or adjunctive treatment strategies may be warranted in PAF cases. Prolonged corticosteroid therapy may help control intrathecal inflammation and reduce spinal cord edema before considering surgical intervention. In addition, approaches aimed at modulating arachnoid fibrosis and improving CSF dynamics may offer benefit. Notably, spinal adhesive arachnoiditis or arachnoidopathy, a human condition characterized by the formation of arachnoid fibrosis and CSF obstruction, is considered analogous to PAF or constrictive myelopathy in Pug dogs (9, 26, 27). Although the underlying pathomechanism remains poorly understood, recent reports in both canine and human patients suggest that a subarachnoid-subarachnoid bypass procedures may help restore CSF flow and alleviate neurological signs (22, 26). Notably, Gomes (19) adapted Meren’s bypass—originally developed for constrictive myelopathy— into a SDS technique for treating SAD. Furthermore, experimental antifibrotic compounds have shown potential in animal models by inhibiting fibroblast proliferation. These include tamoxifen (TAM, a synthetic nonsteroidal antiestrogen) (28), Ro5-4864 (a peripheral benzodiazepine receptor agonist) (29), and pimecrolimus (an ascomycin derivative with immunomodulatory properties) (30). However, the clinical application of corticosteroids, antifibrotic agents, and surgical bypass techniques in dogs with PAF requires further investigation to assess their safety, efficacy, and long-term outcomes.

### 4.3 Do DM, NME, or CSF analysis test results influence the outcome?

Genetic testing for DM was performed on two dogs (cases 6 and 9), with case 6 testing negative for the SOD1 mutation and case 9 being a heterozygous carrier (Table 3). Although a DM diagnosis was considered unlikely in both cases, post-mortem exams were not performed, so the diagnosis remains unconfirmed.

The authors consulted Laboklin® (Manchester, United Kingdom) regarding the genetic distribution of DM in the UK Pug population. Since 2013, approximately 500 Pugs have been tested, with 70% testing clear (negative), 25% being heterozygous (carriers), and 5% being homozygous (affected) for the DM Exon 2 mutation in the SOD1 gene. These findings suggest that the overall prevalence of the risk for developing DM in UK Pug population may be low.

We also consulted Laboklin® (Manchester, United Kingdom) regarding the genetic distribution of NME in the UK Pug population. Since 2013, approximately 2,500 Pug dogs have been tested, with 59% testing clear (negative), 36% heterozygous (carriers), and 5% homozygous (affected) for the NME risk variant on CFA12. A recent study (10) reported that male Pug dogs heterozygous for the NME risk haplotype tended to show earlier onset of clinical signs. Therefore, further investigation is warranted to ascertain the contribution of an immunological etiology to myelopathy in Pug dogs.

CSF analysis was performed in two dogs (cases 1 and 9). Case 1 showed only an increase in protein levels, while case 9 exhibited elevated protein and total NCC. In case 1, the CSF sample was collected cranial to the clinically affected thoracolumbar region, potentially missing the pathological process (31).

Unfortunately, neither dog underwent genetic testing for NME. However, the abnormal CSF findings in case 9 raise the question of whether an inflammatory process may have influenced the clinical outcome and whether prolonged corticosteroid treatment could have been beneficial in this case.

### 4.4 Can sex or age of onset, multiple surgeries, duration of clinical signs influence outcome?

In our study, male dogs were over-represented, consistent with previous reports (1, 5, 7, 9, 10). In cases 3 and 13, longer clinical signs duration and a history of two surgical procedures did not appear to negatively influence outcomes. Case 11 also had a long-standing clinical history but experienced a poor outcome, likely due to the combination of PAF and SAD. Additional factors—including T1–T5 syringomyelia and a corneal ulcer requiring enucleation—may have further contributed to its deterioration (Tables 1, 3).

Notably, case 13 (SAD group), the youngest dog in the study, remains neurologically stable and functional at 9.2 years of age, suggesting that early diagnosis and surgical intervention may improve prognosis, as previously suggested by Skeen (14). However, while Skeen proposed that SAD in young dogs is likely congenital, this may not always be the case, as demonstrated by our case. Therefore, spinal CT should always be pursued to identify the underlying cause of SAD, even in young dogs. In cases where Pug myelopathy is associated with degenerative changes, such as disc protrusion, delays in diagnosis may occur, potentially affecting the outcome.

### 4.5 Does type of vertebral construct or the location, grade, and extension of spinal cord intensity influence outcome?

No apparent relationship was identified between the type of vertebral construct (unilateral or bilateral), the number of stabilized vertebrae (two, three, or four), and clinical outcomes (Table 3).

Follow-up CT scans were available for three dogs (cases 6, 9, and 12), with evidence of mechanical failure identified in case 9. However, without dynamic imaging, vertebral stability could not be definitively assessed, and further diagnostic evaluation was declined by the owner. The small sample size and limited availability of follow-up imaging substantially constrain the ability to draw firm conclusions from these observations. It should be noted that none of the cases in this study received surgical adjuncts (e.g., bone grafts or bone morphogenetic proteins [BMPs]) to promote bony fusion, which may have influenced long-term stabilization and overall outcomes.

Recent studies suggest that the use of 3D-printed drill guides (32) and titanium pedicle screw-rod fixation (33, 34) may improve surgical outcomes by facilitating accurate implant placement and potential revision, while also reducing MRI susceptibility artifacts. Consideration could be given to using these technologies to enhance surgical precision and postoperative assessments in future cases.

In this study, the extent and severity of T2W spinal cord hyperintensity appeared to have a stronger association with outcome than lesion location (gray vs. white matter). This was observed in case 13, where a shorter lesion extension and milder hyperintensity were associated with a favorable outcome. However, further studies are needed to confirm this potential relationship.

Although the combination of T2W hyperintensity and T1W hypointensity has been linked to a worse prognosis due to the presence of severe lesions (e.g., necrosis, myelomalacia, spongiform changes in the gray matter, and white matter necrosis) (35, 36), T1W hypointensity was not observed in cases 9 and 11, where both T1W and T2W sequences were performed. Follow-up MRI was not pursued due to the expected artifact from the metal construct, leaving it unclear whether the clinical deterioration was due to lesion progression or the development of a new lesion.

### 4.6 Do urinary and/or fecal incontinence and UTIs influence the outcome?

Urinary and/or fecal incontinence occurred in five dogs (cases 1, 3, 6, 9, and 11), with recurrent UTIs in four (cases 1,3, 6, and 11; Table 1). While incontinence did not appear to directly influence the overall outcome, it likely contributed to the development of UTIs and, in some cases, added to the caregiver burden associated with progressive neurological decline.

Concurrent disorders such as IBD in case 1 and fecal incontinence in case 6—suspected to be secondary to the thoracolumbar myelopathy—might have also contributed to the UTIs (Table 1). Only four dogs (cases 1, 6, 9, and 11) underwent sensitivity tests, with cases 1 and 6 showing antibiotic-resistant UTIs.

Misdiagnosis of the type of incontinence in cases 3 and 6 resulted in inappropriate initial treatments. Case 6 later developed a urethral obstruction caused by multiple uroliths associated with a UTI, ultimately requiring the placement of a permanent cystostomy catheter to manage detrusor atony. Despite these complications—and the eventual need for a mobility cart—case 6 had the longest survival, largely due to the owner’s remarkable dedication and care.

The findings suggest the importance of closely monitoring urinary incontinence and UTIs in Pug myelopathy. Close collaboration between referring veterinarians and specialists is crucial to avoid long-term complications.

### 4.7 Study limitations

The retrospective design and small sample size of this study limited data consistency and precluded the use of statistical analysis. Follow-up imaging was also limited; spinal CT was available in only three cases, and MRI was not performed due to concerns about metal-induced artifacts. Outcome assessment relied primarily on clinical histories and caregiver reports, which may introduce subjective bias.

Despite these limitations, this study provides valuable insights as the first and only report to date providing long-term follow-up in dogs undergoing vertebral stabilization for Pug myelopathy.

### 4.8 Suggested guidelines for standardized outcome assessment in pug myelopathy cases

Given the current variability in postoperative reporting and the challenges in assessing long-term outcomes in Pug myelopathy cases, there is a clear need for standardized outcome measures. Establishing a consistent framework would facilitate multicenter data comparison, enhance the quality of case series and retrospective analyses, and support future meta-analyses to guide evidence-based decision-making.

Key points for standardized assessment could include:

1. Neurological function, incorporating neurologic grading using a modified Frankel (37) or Olby scale (38), and proprioceptive function (presence and return of conscious proprioception).
2. Functional and quality of life metrics, including assessment of bladder and bowel control (degree and frequency of incontinence) and functional mobility at home (i.e., ability to rise, stability when turning, maintaining posture during toileting, and return to preoperative activity level).
3. Surgical and postoperative outcomes, including radiologic assessment of bony fusion, which may be supported by adjunctive techniques such as bone grafts or BMPs, documentation of postoperative complications (e.g., seroma, dehiscence, infection, implant failure), and time-based recovery milestones (e.g., time to regain ambulation or continence).
4. Long-term follow-up parameters, with recommended reassessment intervals (e.g., 3, 6, and 12 months), and monitoring for outcome stability or neurological deterioration.

Integration of standardized caregiver questionnaires such as LOAD (Liverpool Osteoarthritis in Dogs) (39) via email follow-up could improve data consistency and provide meaningful insights into long-term functional outcomes. Future studies should consider incorporating such metrics to improve comparability and rigor in the evaluation of the outcome in Pug myelopathy research.

In summary, vertebral stabilization combined with decompressive procedures may have contributed to temporary clinical improvement and delayed recurrence of neurological signs in most cases of Pug myelopathy.

However, recurrence was observed within the first year in all but one dog. Spinal CT, used alongside MRI, is essential for identifying VAP dysplasia and may assist in surgical planning. A more favorable prognosis seems to be associated with younger age at presentation, shorter lesion extent, and milder T2-weighted spinal cord hyperintensity, while the presence of PAF may be indicative of a poorer outcome. However, these observations should be interpreted with caution given the study’s limitations.

To explore a potential immunological component in Pug myelopathy, lumbar CSF analysis and genetic testing for NME are recommended. In PAF cases, anti-inflammatory corticosteroid therapy may help manage intrathecal inflammation prior to surgical intervention. Additionally, restoring CSF flow using a subarachnoid–subarachnoid bypass (shunt procedure), in combination with stabilization, could represent a promising surgical strategy. However, further research is needed to clarify the benefits of corticosteroid therapy on intrathecal inflammation, the role of antifibrotic agents in managing arachnoid fibrosis, the efficacy of CSF diversion procedures, and the relevance of NME as a potential risk factor. Urinary incontinence should be carefully monitored and managed to minimize the risk of complications.

Larger, prospective studies with standardized outcome measures and extended follow-up are essential to validate these preliminary findings and guide future therapeutic approaches.

## Data Availability

The original contributions presented in the study are included in the article/supplementary material, further inquiries can be directed to the corresponding author.

## Glossary

BMPs: Bone morphogenetic proteins
CAP dysplasia: Caudal articular process dysplasia
CSF: Cerebrospinal fluid
CT: Computed tomography
DM: Degenerative myelopathy
GFAP: Glial fibrillary acidic protein
IVDP: Intervertebral disc protrusion
LOAD: Liverpool Osteoarthritis in Dogs questionnaire
MRI: Magnetic resonance imaging
NCC: Nucleated cell count
NME: Necrotizing meningoencephalitis
PAF: Pia-arachnoid fibrosis
Pimecrolimus: An immunomodulatory antifibrotic drug
PMMA: Polymethylmethacrylate
Ro5-4864: A peripheral benzodiazepine receptor agonist with antifibrotic potential
SAD: Spinal arachnoid diverticulum
SDS: Subdural shunt (bypass technique)
SIS: Small intestinal submucosa
T1WI/T1W: T1-weighted MRI imaging sequences
T2WI/T2W: T2-weighted MRI imaging sequences
TAM: Tamoxifen, an experimental antifibrotic compound
UTI: Urinary tract infection
VAP dysplasia: Vertebral articular process dysplasia
VCOT: Veterinary and Comparative Orthopaedics and Traumatology (journal)
